# Draft Genome Sequence of *Bacillus* sp. FJAT-27238 for Setting up Phylogenomic Analysis of Genomic Taxonomy of *Bacillus*-Like Bacteria

**DOI:** 10.1128/genomeA.00985-15

**Published:** 2015-09-24

**Authors:** Guo-hong Liu, Bo Liu, Jie-ping Wang, Jian-Mei Che, Qian-Qian Chen, Yu-Jing Zhu

**Affiliations:** Agricultural Bio-Resources Research Institute, Fujian Academy of Agricultural Sciences, Fuzhou, China

## Abstract

*Bacillus* sp. FJAT-27238 is a Gram-positive, spore-forming, and aerobic bacterium. Here, we report the draft genome sequence of *Bacillus* sp. FJAT-27238, with 6,134,829 bp, which will provide useful information for setting up phylogenomic analysis of the genomic taxonomy of *Bacillus*-like bacteria as well as for the functional gene mining and application of strain FJAT-27238. The genomic DNA G+C content was 47.37%.

## GENOME ANNOUNCEMENT

During the course of a study on the diversity of *Bacillus* species from Wudalianchi in Heilongjiang Province of China, strain FJAT-27238, a potentially novel species of the genus according to results of 16S rRNA phylogenetic analysis, was isolated. Recently, it has been proposed that whole-genome sequencing information be combined with the main phenotypic characteristics as a polyphasic approach strategy (taxono-genomics) to describe new bacterial taxa (1–4). In this study, a high-quality genome sequence of FJAT-27238 was sequenced that may confirm that this is a new species and also promote new research regarding the genomic taxonomy of *Bacillus*-like bacteria.

The genome of FJAT-27238 was sequenced with massively parallel sequencing (MPS) Illumina technology. Two DNA libraries were constructed: a paired-end library with an insert size of 500 bp and a mate-pair library with an insert size of 5 kb. The 500-bp library and the 5-kb library were sequenced using an Illumina HiSeq 2500 with a paired-end (PE) 125 strategy. Library construction and sequencing were performed at the Beijing Novogene Bioinformatics Technology Co., Ltd. Quality control of both paired-end and mate-pair reads were performed using an in-house program. After this step, Illumina PCR adapter reads and low-quality reads were filtered. The filtered reads were assembled by SOAP *de novo* (5, 6) to generate scaffolds. All reads were used for further gap closure. Through the data assembly, 6,134,829 bp within 1 scaffold were obtained, and the scaffold *N*_50_ was 6,134,829 bp. Ninety-five percent of the clean reads were aligned back to the genome, suggesting good quality of the assembly.

Gene prediction was performed on the FJAT-27238 genome assembly by GeneMarkS (7). tRNA genes were predicted with tRNAscan-SE (8), rRNA genes were predicted with rRNAmmer (9), and sRNAs were predicted by BLAST against the Rfam (10) database. PHAST (11) was used for prophage prediction and CRISPRFinder (12) was used for CRISPR identification. A total of 5,917 genes were predicted, including 5,583 protein-coding genes, 8 small RNA (sRNA) genes, 125 tRNA genes, and 40 rRNA genes (13 5S rRNAs, 14 16S rRNAs, and 13 23S rRNAs). Also, 6 prophage and 1 CRISPR array were found in the draft genome. The genomic DNA G+C content was 47.37%.

From the genome sequence analysis, FJAT-27238 was predicted to possess complete carbon metabolic pathways, including those for glycolysis and fatty acid biosynthesis and the pentose phosphate pathway. In particular, FJAT-27238 was predicted to be equipped with a wide variety of genes for amino acid and organic acid metabolism, in accordance with the physiological properties of FJAT-27238.

### Nucleotide sequence accession numbers.

This whole-genome shotgun project has been deposited at DDBJ/EMBL/GenBank under the accession no. LGJF00000000. The version described in this paper is version LGJF00000000.1.
